# Non-Linear Pattern Formation in Bone Growth and Architecture

**DOI:** 10.3389/fendo.2014.00239

**Published:** 2015-01-20

**Authors:** Phil Salmon

**Affiliations:** ^1^Bruker-microCT, Kontich, Belgium

**Keywords:** bone, morphogenesis, chaos and non-linear dynamics, architecture, developmental biology

## Abstract

The three-dimensional morphology of bone arises through adaptation to its required engineering performance. Genetically and adaptively bone travels along a complex spatiotemporal trajectory to acquire optimal architecture. On a cellular, micro-anatomical scale, what mechanisms coordinate the activity of osteoblasts and osteoclasts to produce complex and efficient bone architectures? One mechanism is examined here – chaotic non-linear pattern formation (NPF) – which underlies in a unifying way natural structures as disparate as trabecular bone, swarms of birds flying, island formation, fluid turbulence, and others. At the heart of NPF is the fact that simple rules operating between interacting elements, and Turing-like interaction between global and local signals, lead to complex and structured patterns. The study of “group intelligence” exhibited by swarming birds or shoaling fish has led to an embodiment of NPF called “particle swarm optimization” (PSO). This theoretical model could be applicable to the behavior of osteoblasts, osteoclasts, and osteocytes, seeing them operating “socially” in response simultaneously to both global and local signals (endocrine, cytokine, mechanical), resulting in their clustered activity at formation and resorption sites. This represents problem-solving by social intelligence, and could potentially add further realism to *in silico* computer simulation of bone modeling. What insights has NPF provided to bone biology? One example concerns the genetic disorder juvenile Pagets disease or idiopathic hyperphosphatasia, where the anomalous parallel trabecular architecture characteristic of this pathology is consistent with an NPF paradigm by analogy with known experimental NPF systems. Here, coupling or “feedback” between osteoblasts and osteoclasts is the critical element. This NPF paradigm implies a profound link between bone regulation and its architecture: *in bone the architecture is the regulation*. The former is the emergent consequence of the latter.

## Introduction: 3D Architecture in Bone Research

Scientists from many disciplines get drawn into the study of bone architecture. As well as having direct clinical relevance in relation to mechanical competence of the skeleton and fractures, bone structure exerts a fascination – arising perhaps from its functionality, complexity, and even esthetic qualities. A large literature exists on the topic of trabecular and cortical bone architecture. However, the question of how, in the mechanistic developmental sense, bone has the architecture that it does is seldom asked. Why and how does bone travel along its complex spatiotemporal trajectory to acquire its final form? Bone’s architecture has generally been taken as a given – the question that follows is “why is a particular architecture advantageous?” This has been the domain of substantial biomechanical research to date. However, the question “in what way did a particular architecture come about?” and has received far less attention.

In the last decade, micro-computed tomography (micro-CT) has had an energizing effect on research into bone biology, by providing a convenient laboratory based method for imaging bone’s three-dimensional (3D) architecture non-destructively (1). micro-CT has become a routine tool in the preclinical testing of pharmaceutical agents effects on bone. The center of gravity of research at the leading edge of micro-CT technology within bone research, and also that of related technologies such as synchrotron CT and micro-MRI, has been occupied by the field of bioengineering, and the search for answers as to the precise source of structural or architectural failure leading to broken bones.

The engineering performance of the bone, implied from its 3D architecture, is thus one measured endpoint of bone biology experiments. In general, biologists study bone regulation (involving physiological, genetic, endocrine, and cytokine factors) and the bio-engineers study the architecture. This separation into camps fails to address the direct and profound link that, in fact, exists between bone metabolic regulation and its architecture: in bone the architecture *is* the regulation. The former is the emergent consequence of the latter. How is this?

## Non-Linear Pattern Formation

**Figure 1:**
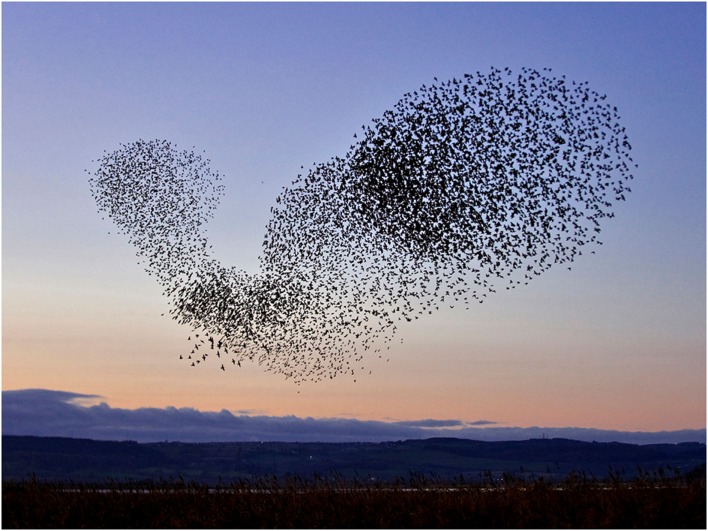
**Pattern-shifting swarms or “murmurations” of starlings are an example of subtle dynamics of communication leading to emergent pattern formation**.

For instance, why is trabecular bone trabecular? This is similar to the question – why do large flocks of birds such as starlings sometimes create moving 3D patterns that are mesmerizing and evocative (Figure 1)? These spontaneous evolving architectures are referred to as “murmurations.” Who or what “tells” each bird where to fly in order to create these beautiful and highly structured 4D patterns? If this were a military pageant, then the answer would be that it is all consciously drilled and rehearsed, micro-managed down to the tiniest movement. But there is no avian “sergeant-major” barking order at individual citizen starlings. So whence the form and pattern?

**Figure 2:**
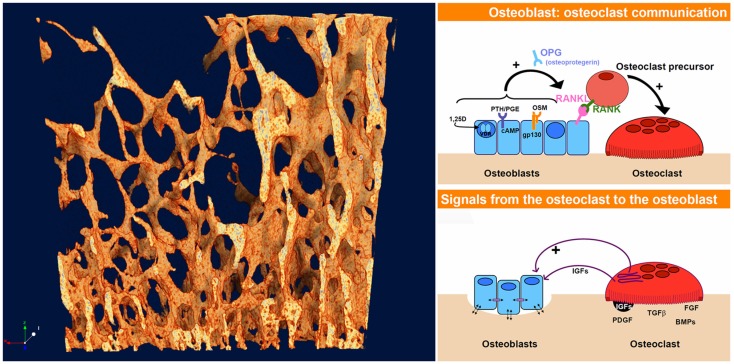
**The dynamics of interaction and coupling between osteoblasts and osteoclasts give rise to the complex evolving 4-D pattern of trabecular bone, just as the responses between starlings give rise to the highly patterned swarming murmurations**. The osteoblast–osteoclast diagrams (right) were taken from an online slideshow provided by the group of Natalie Sims and Ron Martin at the Saint Vincent’s Institute, Melbourne, VIC, Australia [Ref. (4), http://www.ectsoc.org/c020709/sims.pdf].

What unites these questions – how bone gets its architecture and how the shifting shapes of swarming starlings are generated – is that both exhibit spontaneous pattern-formation associated with the dynamics of chaos. This phenomenon, in which structured shapes appear as if from nowhere in dynamic systems is referred to as non-linear pattern formation (NPF) (2). At the heart of NPF is the fact that quite simple rules of interaction between interacting elements in a dynamic system, multiplied and repeated many times, can lead to highly complex, non-repeating, and structured patterns (3). How one starling in a swarm responds to its neighbor, how bone formation by an osteoblast is stimulated by resorption by a nearby osteoclast and vice versa (Figure 2) – the fine scale dynamics of these interaction, multiplied many times over, yield the “emergent” outcome of the evolving shape both of the swarm of starlings and of trabecular bone.

## Coupling and Feedback between Osteoblasts and Osteoclasts

In the same way that birds in a swarm respond to their neighbors, there is increasing evidence in bone remodeling of active communication both ways between osteoblasts and osteoclasts, better described as coupling or feedback (5). The first major feedback link to be found in bone remodeling was the RANK–RANKL–osteoprotegerin (OPG) system (6). Since that discovery, more pathways of coupling between osteoblasts and osteoclasts have been uncovered – several of them appear to be connected with the gp130 co-receptor subunit (7). So active feedback between bone formation and resorption is clearly a reality.

This coupling, alternatively referred to as “feedback,” is an important component of NPF, and thus the discovery of an increasing number of routes of feedback between bone remodeling cells increases the possibility that NPF may play a role in pattern formation in bone. Some of the ways in which this can happen are explored below.

## Role of Osteocytes in Bone Remodeling

In recent years, it is also becoming clear that the osteocyte cells, previously seen as passive passengers in remodeling bone, are themselves active agents of signaling and coordination of the activity of osteoblasts and osteoclasts (8). For instance, advanced “4D” micro-CT analysis of remodeling bone by sequential *in vivo* scanning, combined with image coregistration to identify both forming and resorbing regions, and subsequent spatially resolved biochemical analysis, show, for instance, that RANKL expression is increased specifically within a volume of bone that is shortly to undergo resorption [Ref. (9), personal communication]. Likewise, forming regions were found to have elevated OPG expression. This excellent technical work shows that the important bone signaling molecules operate in a precise spatiotemporal context, and can originate from osteocytes within bone.

## Alan Turing and the “Reaction–Diffusion” Model

**Figure 3:**
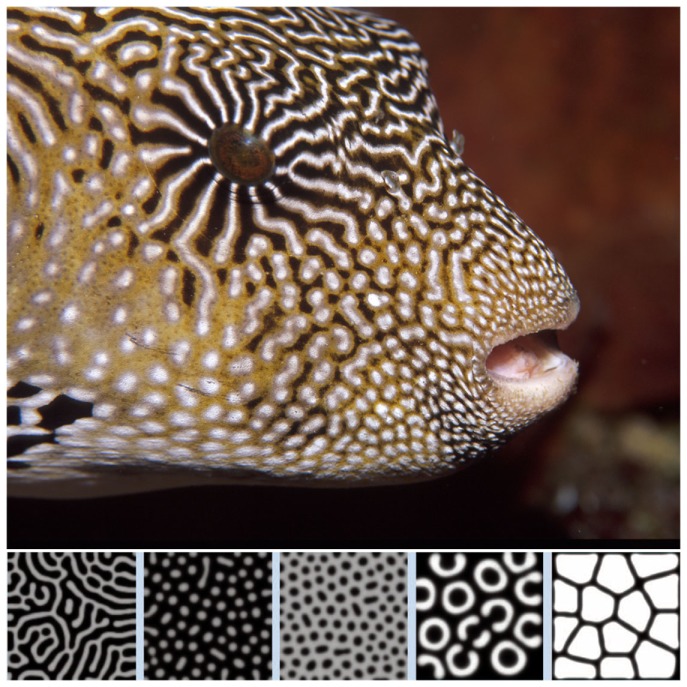
**Turing patterns in biological development**. From Kondo and Miura (11). Reprinted with permission from AAAS. The image of the popper fish is courtesy of Massimo Boyer (www.edge-of-reef.com).

Alan Turing provided a key insight into the regulation of the development of complex biological tissues by his reaction–diffusion model (10), proposed in 1952, essentially an NPF system. The essence of Turing’s model was very simple – two biochemical agents operate in a tissue, one promotes cell growth and operates at short range, the second inhibits growth and operates at long range. Turing demonstrated mathematically that complex patterns emerged from the operation of these simple rules. Examples of Turing patterns are shown in Figure 3. Turing’s insight has proved foundational in biological morphogenesis. Much work since then has confirmed the operation of developed versions of the Turing type reaction–diffusion systems in biological development and morphogenesis (11). For instance, WNT and DKK signaling were shown to represent a Turing reaction–diffusion system in the setting of spacing between murine hair follicles (12). Of course WNT and DKK are well-known players in bone regulation also. Kondo and Miura also referred to the role of “nodal” and “lefty” in left-right asymmetry and that of TGF-beta/FGF and BMPs in tooth pattern, lung branching, and skeletal limb pattern (the *hox* system), as examples of Turing type reaction–diffusion systems.

About a decade after Turing’s paper on the reaction–diffusion model, in the early 1960s, the meteorologist Edward Lorenz, working with one of the earliest computers on the simulation of weather systems, discovered again that a set of simple equations, repeated many thousand times, resulted in complex and non-repeating (“non-periodic”) patterns (13). He further found that the evolution of these complex systems converged mysteriously toward one or more regions of his parameter “phase space.” The term “strange attractor” was later coined for this phenomenon (14). Lorenz’ work is often referred to as foundational in the study of emergent pattern from NPF systems.

**Figure 4:**
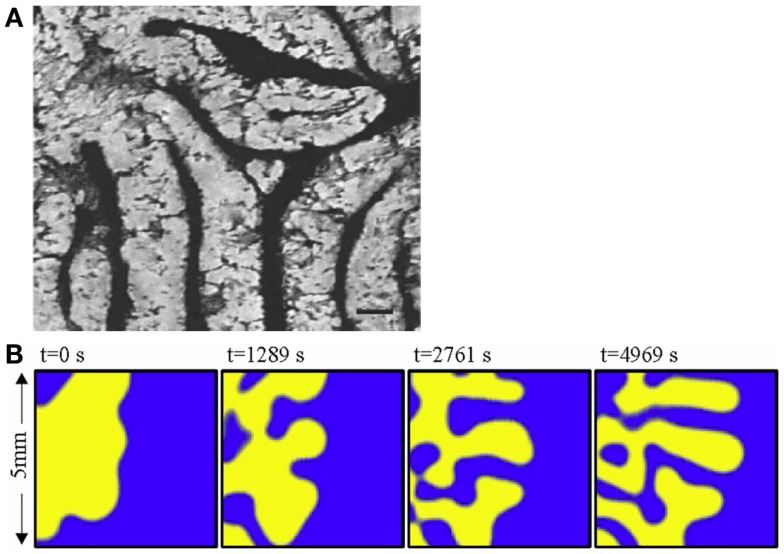
**(A)** The labyrinthine pattern of initial calcification, shown by Yochelis et al. (15) in a culture of mesenchymal cells; and **(B)** the progress of a chemical model of non-linear pattern formation – the Belousov– Zhabotinsky reaction – resembling transition from cortical to trabecular bone at an endosteal surface, by the same author (16). ©IOP Publishing and Deutsche Physikalische Gesellschaft. CC BY-NC-SA. Copyright ©2002 Society for Industrial and Applied Mathematics. Reprinted with permission. All rights reserved.

Turing NPF patterns are visible at the very outset of bone calcification and development. Yochelis et al. showed that the first calcification events, which represent the initiation both of trabecular bone embryonic appearance and also pathological instances of atopic calcification such as in atherosclerotic plaques, are also characterized by labyrinthine Turing patterns [Ref. (15), Figure 4A] representing a reaction–diffusion or promoter–inhibitor morphogenetic system. The same author had previously demonstrated the generation of bone-like labyrinthine patterns with experimental chemical systems (16), such as the classic “BZ” (Belousov–Zhabotinsky) oscillating thin film reaction. The “transverse front instability” or “Ising front” shown in Figure 4B bears resemblance to the process of endocortical trabecularization – the thinning of cortical bone by transition to trabecular architecture at the endosteum, which has become a focus of interest recently in research into age-related osteoporosis at cortical sites such as the distal radius (wrist) (17, 18).

## Turbulence

Once one begins to develop a “feel” for non-linear pattern systems, it is not hard to find them elsewhere in bone structures. For instance, one of the phenomena associated with chaos in fluid dynamics is turbulence. Here is an easy way to see the onset of turbulence. Turn on a tap very slowly. At first, only separated drops of water will fall from the tap. Then a smooth linear flow of water will descend. As you slowly open the tap further, the stream of water will start to wobble and snake from side to side. A little further yet, and the linear stream will break up decisively into a chaotic tumbling cascade. This event is well-known to engineers as the laminar-turbulent transition, or the “turbulent wake transition” (19). Much effort is made by designers to control this phenomenon to reduce wind resistance of cars and improve the flight of aircraft.

**Figure 5:**
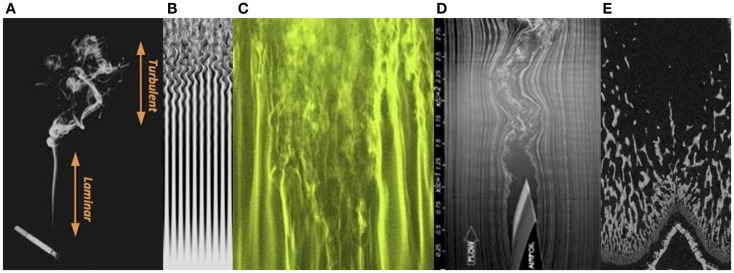
**Transitions from laminar to turbulent flow, in rising cigarette smoke (A), gas flow (B,C), the trailing edge of an aircraft wing (D), and in primary and secondary spongiosal trabecular bone “downstream” of the growth plate in the distal femur of a young rat (E)**.

Images of examples of laminar-turbulent fluid transitions are shown in Figure 5. Among them is an image of a longitudinal micro-CT section through a distal femur of a juvenile rat. The appearance of the primary spongiosal bone immediately “downstream” of the growth plate, in thin, mostly parallel structures, followed a little further from the growth plate by the more complex-chaotic forms of the mature secondary spongiosal trabecular bone, bears resemblance to a laminar-turbulent transition. It is more than just analogy to describe the newly formed bone at the growth plate in terms of a fluid “flowing” away from the growth plate through the metaphysis as the bone extends. In a sense, it is really a highly viscous and slow moving fluid. The bone’s architecture changes hour by hour, day by day, and a parcel of bone formed at the growth plate could be millimeters away from it a week later.

Turbulence represents the full onset of chaos, and the complex 3D structure of turbulent flow can be described as a chaotic cellular structure. This might well be a fitting description of trabecular bone, as the spatiotemporally separated formation and resorption loci transform the bone’s architecture in four dimensions, essentially unconstrained in space. The visual likeness of the long bone metaphyseal trabecular bone to a flowing fluid developing turbulence provides a hint as to the underlying pattern-forming process.

## NPF Gets Social – Particle Swarm Optimization

We can return to the subject of the spontaneous patterns of the swarming birds (Figure 1). The study of these far-from-random evolving 3D shapes of bird swarms and fish shoals, has led to a theoretical model representing a form of NPF, called “particle swarm optimization” or PSO (20). This is related to a body of theory called artificial life or “A-Life.” Birds such as starlings which form large flocks are observed to “flock synchronously, change direction suddenly, scatter and re-group iteratively, and finally perch on a target” (21). PSO increases the success rate in foraging for food targets by a bird swarm, or a shoal of fish, and is referred to as “social intelligence” (21). The algorithm of the PSO model simulates quite simple interactions between “particles” (e.g., birds) that include a stochastic (random) component, an adaptive component and positive feedback, in fact, recognizable elements of NPF.

Without getting into any of the actual maths, the elements of a PSO model can be outlined (21).

### Solution space

The solution space is a volume within which a location has to be found that meets criteria for being “optimum.” For instance, the volume of air in which the birds are swarming.

### Parameters

There are one or more parameters characterizing each location in the solution space.

### Problem

There is a problem to be solved which requires finding the location, within the solution space where one or more parameters have optimal values.

### Particle

The particle exists at a vectorial location within the solution space, and iteratively explores the solution space. The particle senses and registers the value of the parameters at each location that it visits.

### Swarm

The swarm is a large group of particles in the solution space. Each particle in the swarm moves at a certain velocity. The particles, as they move around, search for the optimum solution to the problem by referring to previous experiences.

### Particle’s best experience

Abbreviated to *pbest*, this means the “best” experience of an individual particle, based on the parameter values.

### Particle’s neighbor’s best experiences

Abbreviated to *lbest*, or local best, this is the best experience of any of a particle’s neighbor particles, up to, for instance, two particles away.

### Swarm’s best experience

Abbreviated *gbest*, or global best, this is the best experience had by any particle in the swarm.

### Particle movement

The movement of each particle is partly random, but is also partly influenced by the history of experiences of both that particle and its near neighbors.

### Algorithm

The particle moves its position in response to both its own experience and the “swarm intelligence.” This is represented by a mathematical weighting toward locations corresponding to both *pbest* and *gbest*.

This PSO model calls to mind a city center on a Friday night full of people looking for the best night club or party venue, comparing experiences of the best DJ’s or the worst bouncers, and homing in on the locales where the best time is to be had.

It turns out that the “social influence” or *lbest* is critical to the performance of the swarm (22). Remove *lbest* and the swarm’s effectiveness in solving the problem sharply decreases.

The phenomenon of an “attractor” is a feature of chaotic NPF – a structured subset of a system’s phase space to which system elements converge. In the chaos literature it is sometimes referred to as a “strange attractor.” In PSO models, particle swarms also converge toward an attractor (22). Note that in a NPF system including a PSO model both the phase space and the attractor can be multi-dimensional. Multidimensionality is relevant in a biological cell and tissue context due to the large number of signals and influences that a cell will experience.

Can individual cells, as well as organisms like birds, display “social intelligence” of the PSO type, involving adaptive behavior and interactions and information exchange with neighbors? Evidently they can – bacteria are a well-studied example exhibiting optimized group foraging strategies following PSO or similar models (23–25).

In bone remodeling, could it be that osteoclasts and osteoblasts act as a particle swarm, showing PSO behavior, as they respond to multiple signals operating at different ranges? Could this explain why, for instance, remodeling operates at discreet, concentrated locations – the bone remodeling unit (BMU) elucidated by Frost (26), rather than spreading out more uniformly or randomly on bone surfaces?

Furthermore, it turns out that the 3D architecture of bone marrow is important in cellular participation in bone remodeling. Looking at often-presented signaling cartoons, it is easy to imagine that these processes take place within a uniform featureless soup, or like atoms within a well-mixed gas. Evidence that this is not so was given by de Barros et al. (27) who showed that “migration, proliferation, and differentiation of hematopoietic stem cells (HSCs) are dependent upon a complex 3D bone marrow microenvironment.” Thus, as well as endocrine and cytokine actors, there is also 3D landscape involved in bone cellular metabolism. This issue of microenvironment 3D architecture has become recognized as important in the field of regenerative medicine and the fabrication of osteogenic scaffolds for bone tissue repair, where all factors need to be understood which impact on the success of ingrowth of bone progenitor cells into bone repair scaffolds (27, 28).

One can imagine PSO operating between groups of osteoblasts as they negotiate hemopoietic bone marrow environments “looking” individually or collectively for suitable sites to set up a bone modeling unit. Such a paradigm would require evidence that cell populations such as osteoblasts communicate mutually to each other, as well as to other cell populations; such evidence is given by Ziambaras et al. (29); Grellier et al. (30); and Santos et al. (31).

But if we speculate that bone remodeling cells engage in PSO, how could osteocytes, physically locked in their bone lacunae, show swarming behavior? To envision this, we must remember that spaces exist other than the familiar 3D one. Mathematicians use the term “phase space” to describe a system in which each variable parameter is assigned a dimension of its own. Thus, for instance, the expression by osteocytes of RANK, RANKL, OPG, and other signaling molecules could each be assigned a dimension. The full profile of signal expression by an individual osteocyte could be represented by a location in the resulting multi-dimensional phase space (here, one can have as many dimensions as you want). In such a space, our osteocytes are free to soar and swarm like the starlings discussed earlier, under influence of neighbors, even while locked spatially within their bony lacunae.

## Applications for the NPF Paradigm in Bone Biology

What has the paradigm of NPF as a driver of bone architecture provided to bone biology, in terms of insights into bone pathologies? One striking example concerns the rare genetic disorder known both as juvenile Pagets disease (JPD) and as Idiopathic Hyperphosphatasia. This consists essentially of a mutation partially or fully inactivating the gene for OPG. OPG is well known as a key component of the RANK-RANKL system of regulation of bone remodeling, OPG being a competitive inhibitor of the signaling to RANK by RANKL. Predictably, withdrawn of this inhibitor or “damper” of remodeling coupling/feedback results in “runaway” remodeling, much increased remodeling rate and severe ensuing osteoporosis (32).

**Figure 6:**
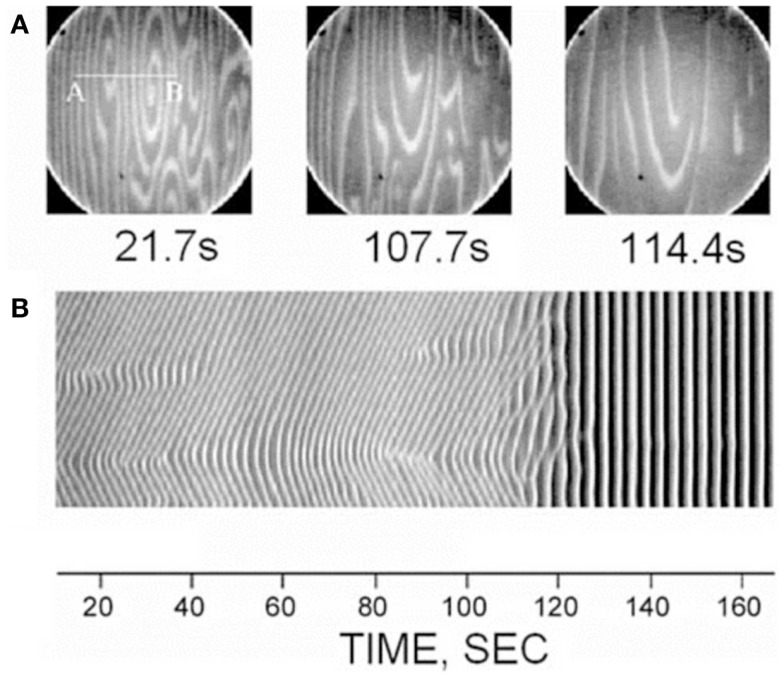
**A direct observation of the suppression of non-uniform patterns by feedback in the platinum-catalyzed CO oxidation reaction, as viewed by photoemission electron microscopy on the platinum Pt(110) crystal surface (34)**. Dark and light areas in the images correspond to regions predominantly covered with oxygen and CO, respectively. The top row **(A)** shows three image snapshots at different times, and the bottom row **(B)** shows space-time diagrams along the line AB indicated in the first image. Reprinted from Pollmann et al. (34). Copyright ©2001, with permission from Elsevier.

**Figure 7:**
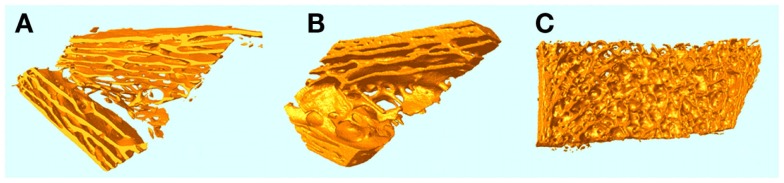
**micro-CT 3D images of iliac crest trabecular bone biopsies from a JPD patient before (A) and a year after (B) treatment with bisphosphonate to reduce the elevated rate of remodeling**. A normal iliac crest biopsy is shown for comparison in **(C)**.

A curious aspect of the bone pathology of JPD shown by Cundy et al. and others by histology is an abnormal parallel arrangement of trabecular plates in iliac crest biopsies, with trabecule aligned in parallel with the iliac cortical wall. Salmon (33) proposed that this parallel trabecular architecture represented the consequence of the removal of damping from resorption–formation coupling or feedback. A physical–chemical analogy to this is the platinum-catalyzed oscillatory oxidation of carbon monoxide on a platinum surface, in which the normally chaotic spiral patterns of oxidation are transformed into an array of parallel lines by the increasing of feedback in an experimentally controlled system (34). This is shown in Figure 6. By comparison, 3D micro-CT images of iliac crest biopsied trabecular bone are shown in Figure 7 for a JPD patient before and after bisphosphonate treatment (35). The parallel pattern of trabecule is quite striking in the JPD case. In both the JPD trabecular bone and the CO oxidation on platinum, increasing feedback “kills” the chaotic pattern and imposes parallel regularity. Thus, the role of OPG in bone remodeling, as an inhibitor or “damper” of feedback/coupling, can be seen as essential in preserving the normal chaotic architecture of trabecular bone. Its withdrawal can lead to the pathological parallel trabecular pattern in JPD.

Mechanically the parallel trabecular architecture is disastrously weak, as evidenced by the breaking into two halves of the biopsy sample (Figure 6A) prior to embedding. Bisphosphonate therapy only partially restored a more normal chaotic trabecular pattern in the central region of the iliac crest, while the trabecule near the cortices remained aligned in parallel with the cortex. Thus, while bisphosphonate slows down the turnover rate (accelerated by JPD) and thereby achieves some limited mitigation of the abnormal architecture, it cannot fully substitute the feedback-damping effect of OPG.

Aside from the exceptional case of JPD, a perspective of NPF establishes a direct link between trabecular architecture and the coupling of remodeling at the level of individual sites of osteoblast formation and osteoclast resorption.

To put it another way, the absence of a pattern-forming perspective in bone research leads to assumptions regarding the link between bone formation and resorption and bone architecture that are inappropriately simplified. An example of this is the belief that increased bone formation rate should lead always to increased trabecular thickness – and inevitable ensuing confusion in interpreting some trabecular morphometric data where this is not the case. The key point is that trabecule cannot be envisioned as static structures, so that the effects of net formation and resorption can simply be added and subtracted arithmetically from surfaces to predict an outcome in thickness. Instead, they are dynamically folding and reshaping their architecture according to emergent non-linear pattern arising from the dynamics of interaction between the agents of bone addition and subtraction – the osteoblasts and osteoclasts. Therefore, data on mere quantitative change to formation and resorption tells you little about the architectural change.

Osteoporosis drugs are often characterized as “anti-resorption” or “anabolic.” However, it is impossible to target formation without affecting resorption, and vice versa, since the two are known to be bi-directionally coupled. Through NPF, the spatiotemporal pattern of change to osteoblast and osteoclast activity and the change to coupling dynamics translates to a complex and not easily predictable change in 3D architecture.

Thus, the “message” given to bone by the gene product or drug or changed mechanical stimulus is not simply a quantitative signal – “get more bone” or “get less bone,” but via the agency of NPF becomes a shape or morphological signal – “go to this shape.”

In terms of the practice of trabecular bone morphometry, by histology or micro-CT, for instance, the following implications arise from the NPF perspective:

- Changes to trabecular thickness, separation, and number are not easily predictable from quantitative changes to formation and resorption;
- Changes to trabecular architecture might result where quantitative changes to formation and resorption are very small or undetectable, resulting from spatiotemporal changes and/or changes in formation–resorption coupling;
- Certain parameters might be useful to refer to regarding the nature and complexity of trabecular architecture, such as fractal dimension (36), trabecular pattern factor, structure model index, and connectivity;
- Make sure that volumes of interest that are analyzed for trabecular bone are large enough to capture changes in spatial patterns and possibly gradients.

In terms of drug discovery for bone medicine, there are also implications. Principally, it is not necessary to restrict the search of gene targets or drug candidates to ones that will affect bone formation or resorption only quantitatively. More subtle effects on formation–resorption coupling or on the spatiotemporal pattern of osteoblast and/or osteoclast action could also be looked for, which might prove therapeutic even in the absence of a clear quantitative change in bone formation or resorption. Indeed, some studies have found a weakness in the relationship between fracture prevention efficacy of a bone drug and the corresponding reduction in bone mineral density (BMD), a surrogate for the spatial density of trabecular structures (37), and it has been demonstrated that change to bone remodeling rate by itself, independent of the agency of change to BMD, can lower fracture rate (38, 39). Speculation as to the mechanism for this has referred to change to the number of erosion sites (“stress risers”) at the bone surface affecting the bone’s strength. An alternative possibility, in the light of NPF and the relationship between bone turnover coupling and bone architecture, is that a change in the rate and other characteristics of bone remodeling caused by a therapeutic agent might change the bone’s 3D architecture in a way favorable to its mechanical strength. Note that the highly pathological parallel trabecular architecture found in JPD patients, as discussed above, is associated with sharply accelerated bone remodeling.

## Bone Morphology, Mechanical Loading, and NPF

The mechanical aspect of bone morphogenesis should not be overlooked of course – bone’s architecture is ultimately determined by the demands of loads and torques it is required to resist, according to Wolff’s law (40). The primary role of bone is a mechanical one, to provide strength and rigidity and to resist with a margin of safety the mechanical loads encountered in an animal’s activities. It has been shown that aspects of bone architecture can be modeled as self-organization in response to mechanical loading (41).

However, if the interactions between formation and resorption include a mechanical term, this will not stop them from being non-linear and thus yielding complex-chaotic emergent pattern when integrated many times over many locations. This helps us understand the difference between nature’s designs and our own. To make structures to meet certain engineering requirements we would use H-beams, rectangular plates, round rods, or square meshes, while nature makes chaotic trabecular bone with regional predominant orientations to reflect load directions. We think linear, nature thinks non-linear. In fact, a mechanical component to the “algorithm” determining formation–resorption interactions is likely to be highly non-linear, because all incremental bone growth and remodeling will change the loading environment. And this is the essence of non-linearity that system parameters themselves are changed by the evolution of the system, as was nicely articulated by Gleick (42) in his (highly readable) book “Chaos”: “non-linearity means that the act of playing the game has a way of changing the rules.”

## Summary

To summarize, the idea proposed here is that in bone “the architecture is the regulation.” Through the agency of the laws of chaotic NPF, the dynamics of interaction at the smallest level between units of osteoblast formation and osteoclast resorption, multiplied in space and time, give rise to the elaborate patterns of bone, such as trabecular bone, as what is described in chaos terminology as an “emergent” phenomenon. Such pattern phenomena form an important element in developmental biology, and more widely in many spatiotemporal patterns observed in nature. There is also the possibility that cell populations such as osteoblasts, osteoclasts, and osteocytes might act “socially,” responding to mutual signals in order to coordinate their group behavior more optimally, according to the theory of PSO. This perspective needs to be included in the interpretation of bone morphometric observations of the effects of agents of change in bone such as drugs or altered genes. It also focuses attention on the nature of coupling between bone formation and resorption as a fundamental factor directly influencing the architecture of bone.

How could NPF in bone metabolism be tested? The idea of PSO in bone remodeling, for instance, could be investigated by looking for evidence of communication between neighboring cells of the same type – osteoblasts, osteoclasts, and osteocytes. And “*in silico*,” computer simulation could aim to reproduce phenomena such as a laminar-turbulent wake transition “downstream” of the growth plate, and transformation between trabecular and cortical bone at endocortical boundaries (trabecular to cortical during bone fetal development and fracture callus formation, the reverse during osteoporosis).

This article falls short of scientific rigor in the sense of logical and experimental proof of this proposal. Instead, a paradigm is proposed, and bone researchers are invited to look for ways in which it might add to the understanding of phenomena of bone architecture observed clinically or experimentally.

## Conflict of Interest Statement

Phil Salmon is an employee of Bruker-microCT.
